# Comment on Huang et al. Influence of Varied Dietary Cholesterol Levels on Lipid Metabolism in Hamsters. *Nutrients* 2024, *16*, 2472

**DOI:** 10.3390/nu17121944

**Published:** 2025-06-06

**Authors:** Leslie M. Klevay

**Affiliations:** Internal Medicine, School of Medicine and Health Sciences, University of North Dakota, 1301 North Columbia Rd., Grand Forks, ND 58202-9037, USA; leslie.klevay@ndus.edu; Tel.: +1-701-772-6960; Fax: +1-701-777-4490

Huang et al. [1] fed groups of Syrian hamsters diets with graded doses of cholesterol for eight weeks. As expected, plasma cholesterol increased from a control value of 135 to 422 mg/dL for animals fed 1% cholesterol. Hepatic glutathione also decreased by 26%. The authors allude to antioxidant defense and oxidative damage, etc. Glutathione may affect these processes related to cardiovascular disease.

Copper deficiency induced by cholesterol feeding has been confirmed several times in four independent laboratories [2] since its discovery more than three decades ago [3]. Wei et al. [4] produced copper deficiency in mice that was verified by decreased cardiac superoxide dismutase (SOD) and serum copper. Cardiac glutathione also decreased; this finding resembles the decrease in hepatic glutathione found by Huang et al. [1].

It may seem odd that lipid peroxidation in the liver was decreased [1] if copper deficiency was induced. A comparison of metabolic changes in several evaluations of copper status in animals and people revealed a wide range of changes: 11 to 94% [5]. Perhaps lipid peroxidation is comparatively resistant to deficiency [6].

Kuhn [7] suggests that when scientists fail to recognize and control a relevant variable, experiments must be performed again. Perhaps the authors [1] will repeat their experiments with an extra group fed increased dietary copper to determine which of their findings depends on adequate copper. Measurements of copper status such as ceruloplasmin, serum copper, and SOD will be helpful. Ceruloplasmin may be a less sensitive measure of copper deficiency than SOD [5,8], which may play an important role in shielding intracellular components from oxidative damage [9].
